# Drug use, harm-reduction practices and attitudes toward the utilisation of drug safety testing services in an Irish cohort of festival-goers

**DOI:** 10.1007/s11845-021-02765-2

**Published:** 2021-09-20

**Authors:** Jo-Hanna Ivers, Nicki Killeen, Eamon Keenan

**Affiliations:** 1 Public Health & Primary Care, School of Medicine, Trinity College Dublin, Dublin, Ireland; 2 Health Service Executive, National Social Inclusion Office, Dublin, Ireland

**Keywords:** Drug checking, Festival drug use, Harm reduction, Pill testing, Sexual health

## Abstract

**Background:**

Festival drug-related deaths are a growing public health concern.

**Aim:**

To examine drug use and related harm-reduction practices and attitudes towards utilisation of drug safety testing services.

**Methods:**

Data collection took place over the 2019 festival season (June–October). The questionnaire was self-reported. Data was gathered via the online survey, which was promoted through online and social media platforms and outlets. Social media communication methods were used to reach the targeted population more effectively.

**Results:**

A total of 1193 Irish festival attendees over the age of 18 completed an anonymous online survey. Alcohol, MDMA powder/crystals, ecstasy pills and cocaine were the highest reported drugs used by Irish festival attendees. The vast majority of participants reported polysubstance use (86.8%/*n* = 1036). Forty percent of participants (39.98%/*n* = 477) reported having had sex following the use of a drug at a festival; of these, 66% (*n* = 316) said that the sex was unprotected. Most participants (84.0%/*n* = 1003) engaged in some form of harm reduction when taking drugs at festivals.

Overwhelmingly, participants reported a willingness to engage with drug-checking services. The vast majority (96.3%; *n* = 1149) and would use drug checking services more than three-quarters (75.1%/*n* = 897) reported that they would use an ‘amnesty bin’ for drugs if it were part of an alert system to notify if dangerous drugs are in circulation. A chi-square test of Independence was conducted to examine whether age and utilisation of drug safety testing service a festival were independent. Moreover, when all cases are taken together, the difference between testing modalities (onsite, offsite and amnesty bin) shows a significant difference *p* < 001 between those who would use onsite and offsite drug testing facilities.

**Conclusion:**

The evidence from this survey indicates that those young people who use drugs at festivals would be prepared to utilise drug checking services and amnesty bins should help inform the public health response to this important area.

## Introduction

Festival drug-related deaths are a growing public health concern [1]. At present, there is limited evidence highlighting drug trends and harm-reduction practices among Irish festival attendees. People who use drugs at festivals and in nightlife scene generally form part of the socially integrated youth culture. Music festivals have grown in numbers and size over the last decade throughout Europe, attracting a diverse range of people whose patterns and experience of substance use varies. Use in nightlife settings can be risky with people changing their pattern of use for the duration of an event, with occasional ‘recreational use’ becoming daily use, frequent and part of a poly pattern [2]. While studies on substance use in nightlife environments generally focus on young people in their own countries, nightlife or ‘experience’ tourism is growing. Young Europeans travel abroad each year to party in nightlife-focused holiday resorts, festivals or on city breaks. Levels of drug and alcohol use and associated risk-taking behaviours often increased during these periods [3, 4].

One of the main health concerns for this cohort relates to the risks associated with high volumes of drugs being consumed as well as the combinations of substances used together. Combining substances can involve the person actively seeking desired effects from the mixture to suit the setting. For example, because of the overall sedative effects of ketamine, some may combine it with stimulant drugs such as cocaine or ecstasy [5]. In addition to the health risks generally associated with drug use in the nightlife settings, it is also known that individuals who engage in higher levels of drug consumption and those who regularly attend clubs and festivals practice riskier sexual behaviours [6]. Lim et al. (2007) collected sexual health data from over 900 individuals who attended Australia’s Big Day Out music festival in 2005. The authors found that 30% of the participants who engaged in sexual activity within the last year either did not use condoms ‘at all’ or did not use them ‘most of the time’ [7]. These individuals were classified to be at high risk for sexually transmitted infections (STIs). However, 24% of those identified as high risk did not consider themselves to be at higher risk of contracting an STI when compared to counterparts who did use condoms. Of the sexually active individuals in the survey, 43% shared that they had not used condoms in the past due to intoxication by alcohol or other substances. No sexual health data exists on festival-goers and or those engaged in the night-time economy. Moreover, advice and resources provided in these setting in Ireland are haphazard and minimal.

For some time, harm-reduction interventions within the night-time economy in Europe have included drug-checking services. Drug-checking services provide members of the public with the opportunity to submit a psychoactive drug for content analysis anonymously. The facility can then provide individual feedback on what the sample contains and the health and safety risks of the substance [8]. Drug-checking services employ two distinct systems: fixed-site services or onsite services. Fixed-site services have a permanent location and laboratory where consultations may take place. In contrast, onsite services are set up at an area of interest, such as a music festival or nightclub, for the duration of an event [8]. Once the analysis is complete where contaminants are found if necessary, warning alerts are issued to attendants. For a review of drug checking systems, see Burnt et al. (2017).

Although drug checking has been trialled internationally, with demonstrated value as a harm-reduction and health-promotion strategy, the use of such services remains a contentious issue. There is some suggestion that drug checking may have adverse consequences; for example, it has been argued that such interventions may encourage the normalisation of drug use or provide a false sense of security. However, these claims are not corroborated by any scientific evidence available to date [9]. Moreover, Brunt challenged the criticism that drug testing encourages young people to take drugs or to increase their use and determined the criticism to be unfounded; indeed, drug use does not appear to increase following the introduction of a drug-testing service [10, 11]. Evidence does not support the view that offering a drug‐checking service at a festival will result in drug use by people who have never used drugs nor does it support the view that a drug‐checking service will lead to increased use among people who use drugs [12, 13].

Olsen et al. (2019) evaluated a drug-checking service in Australia. The participants reported a positive experience with the service [9]. Day et al. (2018) collected data from over 650 music festival-goers in Australia. Of all participants, 86.2% agreed that free drug-checking services ought to be provided at music festivals, with 67.5% agreeing that if they were not offered for free, they should still be available at a cost. Barratt et al. (2018) conducted a similar study with the intent of identifying the parameters in which a drug-checking service could be provided to consumers. They found that most participants would use both onsite (94%) and fixed-site (85%) drug-checking facilities. Moreover, most participants (68%) were willing to pay a small sum for drug-checking services, and one-third were willing to donate a full dose for testing [8].

While research capturing drug users’ attitudes towards drug-checking facilities is lacking in Ireland, the UK has run trials examining both onsite and fixed-site drug-checking services [14, 15] for festival and club-goers. Measham (2020) conducted an onsite testing trial on five dates across three venues in two cities in the UK; in addition to identifying substances submitted, results showed that the participants engaged in harm-reduction practices such as warning other drug users (37.5%), being more careful when engaging in polysubstance use (35.4%) and taking a lower dose (27.8%). Lower numbers reported disposing of the remaining substance (6.9%) and committing additional substances for further testing (2.8%). While the service requires further research before widespread implementation can be considered, the results suggest that service users take the feedback seriously and are willing to engage in harm reduction through drug-checking services.

Moreover, Ireland’s current Drug Strategy Reducing Harm; Supporting Recovery highlights the need for targeted harm-reduction, education and prevention measures and tailoring initiatives for higher-risk groups, such as people who use drugs in settings where drug-taking is common (e.g. festivals). Harm-reduction initiatives and drug welfare are becoming the mainstream at festivals in many European countries, and there have been similar initiatives in Ireland. However, there is no data on the prevalence of drug use and the harm-reduction practices in Irish festival-goers.

To our knowledge, this is the first review specific to festival drug use and harm reduction in Ireland to measure the latest drug trends, related behaviours and attitudes towards drug-checking services. This work found that the provision of drug identification services could support surveys to ensure the accurate reporting and monitoring of emerging drug trends in nightlife settings while also informing early harm-reduction responses.

## Aim

The current study aims to examine drug use and related, harm-reduction practices and attitudes towards utilisation of drug safety testing services.

## Materials and methods

The study surveyed individuals living in Ireland about, drug use, harm-reduction practices and attitudes towards drug-checking services. Data collection took place over the 2019 festival season (June–October). The questionnaire was self-reported and focused on the proceeding 12 months. The questionnaire required between 5 and 10 min to complete.

The majority of questions focused on multiple choice, though, we did provide a small number of text boxes for respondents to expand answers; however, as these were utilised by less than half of participants and the response differences varied significantly, these questions have not been included in the current paper.

Data was gathered via the online survey, which was promoted through social media platforms and outlets.

## Participants

The study targeted people residing in Ireland who consumed substances at music festivals in the 12 months preceding the survey either in Ireland or abroad. Participants between the ages of 18 and 34 were encouraged to participate as drug use levels tend to be higher among this cohort. The survey was an anonymous online survey hosted on the Smart Survey Website.

### Recruitment

Recruitment strategies included several approaches, mainly focusing on channels where young populations, people who use drugs and those interested in nightlife activities and dance music could be targeted. The approach was mainly organic despite one small partnership with Four Four dance magazine.

### Festival harm-reduction campaign media launch

The survey was launched as part of the Health Service Executive (HSE) Festival harm-reduction campaign in 2019. This was a multi-component approach involving a media release, new resources for festivals, outreach and training for festival medics. As part of this launch, the survey was featured in mainstream media publications as well as niche publications such as Gay Community News. As part of the campaign promotion, the survey was discussed across media channels on the radio (radio 1), print (The Journal.ie and The Irish Examiner) as well as on TV (Prime Time, RTE 1). It is anticipated that a diverse audience was captured through this method. An easy hyperlink was established so people could quickly the details at drugs.ie/festivals.

### Online and social media promotion

Online promotion predominantly focused on Drugs.ie communication channels which included the Drugs.ie website, Drugs.ie Twitter (estimated 9500 followers in 2019) and Facebook (estimated 19,000 followers in 2019). These posts were organic without investment in paid adverts. These channels mainly target young populations who use drugs and addiction professionals. Posts were promoted by HSE accounts, well-known youth representatives, Drugs.ie followers, drug services, Drug and Alcohol Task Forces, politicians, student services and the Union of Students in Ireland. A number of posts were shared during and after the festival season with the survey closing after the Boxed Off Festival at the end of September.

### Partnership with ‘Four Four’ dance music magazine

The Irish dance music magazine Four Four shared information about the survey on their site, through their Facebook and Instagram accounts as well as within a private discussion group for their followers. Their audience would predominantly be young cohorts who frequently attend nightlife venues and festivals (e.g. https://fourfourmag.com/take-part-in-the-hses-study-about-drug-use-and-harm-reduction-at-festivals/).

### Promotion at Boxed Off dance music event

As part of education and harm-reduction resources at the Boxed Off dance event, information was available about the survey. Those who engaged with the HSE information stall at this event were encouraged to take part in the survey.

Although some packages can capture if participants click onto the survey Smart Survey does not have the capturing feature. Thus, it is not possible for us to estimate the reach of the social posts, or by site links as this does not guarantee participants clicked onto the Survey. We as we did not have links tracked.

From what is available from the download of data at the time we know that 1193 answered. On review of dates, we jumped from 788 to 1193 from the 26th of September onward which is linked with partnership with Four Four Magazine from the 26th onwards. From June 18th to September, we had 513, showing that promotion worked better after festival season was coming to an end during September. It is reasonable to assume that the survey has substantial input from the Four Four partnership. We only capture full responses of survey.

## Ethical approval

Ethical approval was granted by the Ethics Committee, School of Medicine, Trinity College Dublin.

### Analysis

Data were analysed using SPSS.

## Results

A total of 1193 Irish festival attendees living in Ireland over the age of 18 completed an anonymous online survey. Targeted communication methods offered an advantage in reaching the population more effectively. People who use drugs at festivals have been identified as a hard-to-reach population who may never present to traditional addiction services in Ireland.

Respondents were between the ages of 18 and 34 years old. The average age of participants was 24 years old, 54.2% identified as male, 46.3% identified as female and 0.4% identified as other. Drug use was a significant part of the festival experience, with 96% of the current cohort reporting drug use at festivals in the proceeding 12 months. The survey examined trends in drug use, harm-reduction practices and willingness to engage with drug-testing services. The survey ran for 5 months (from June 2019 to October 2019); the period of interest was 2018 to 2019.

The majority of respondents identified as either employed or studying full time. Music preference varied greatly; however, techno was the single most cited genre, mentioned by more than one-fifth of participants (21.9%/*n* = 262).

Over half of participants (52.9%/*n* = 632) had attended one or two festivals in 2018. Similarly, more than half of participants (54.5%/*n* = 651) intended on attending one or two festivals in 2019.

Participants travelled extensively to attend festivals with 52% reporting the use of drugs at a festival abroad. A total of 20 different countries across three continents (America, Asia and Europe) were cited. When asked for additional comment on using drugs abroad, several respondents indicated that their experience was ‘much stronger than at home’, that is of a higher potency drug compared to what they consume in Ireland.

Alcohol 96.9%, MDMA powder/crystals 84.9%, cocaine 81.8% ecstasy pills 80.3% and were the most commonly reported drugs used by Irish festival attendees with less than 2% reporting the use of dimethyltryptamine (DMT) and gamma-hydroxybutyrate (GHB). Table 1 illustrates the substances used at festivals in the last 12 months. The vast majority of participants reported polysubstance use (86.8%/*n* = 1036)—that is, using two or more substances at once. On average, the participants reported using three substances at any one time (minimum of two and a maximum of eight). The most commonly cited combinations involved alcohol, cocaine, ketamine and MDMA. Participants were asked to report on substances they had consumed at a festival, either in Ireland or while abroad. In the main, participants, substance use was similar; however, they reported disproportionately higher use of mushrooms, 2 CB, DMT, nitrous oxide and mephedrone while attending festivals abroad. The trend ‘CK’ or ‘Calvin Klein’ was mentioned by a small number of participants indicating the intentional combination of cocaine and ketamine [16].

**Table 1:** Substances used at festivals in the last 12 months (Ireland and abroad)

Substances used at festivals in the last 12 months	Ireland *n* = 1194	%	Abroad *n* = 619	%
Alcohol	1157	96.9	606	97.9
Cannabis/weed	853	71.5	467	75.4
Mushrooms	174	14.6	142	22.9*
2CB	313	26.2	208	33.6*
DMT	21	1.7	14	2.26
Ecstasy	959	80.3	554	89.5
Cocaine	977	81.8	542	87.6
Amphetamine speed	204	17.1	145	23.4
GHB	20	1.6	16	2.6*
NPS	30	2.5	23	3.7*
Nitrous oxide	336	28.16	232	37.5*
MDMA	1008	84.49	556	89.8
Ketamine	756	63.37	444	71.7
LSD	252	21.12	172	27.8
Mephedrone	38	3.19	33	5.3*
Benzodiazepines	142	11.90	84	13.6
Unknown pills	98	8.21	63	10.2
Unknown powders	91	7.63	56	9.0

Due to rounding errors, percentages may not equal 100%

^*^Significant changes between substance use at home and abroad

Forty percent of participants (40%/*n* = 477) reported having had sex following the use of a drug at a festival; of these, two-thirds (66%/*n* = 316) said that the sex was unprotected. Table 2 illustrates sexual health services utilised by participants at festivals.

**Table 2:** Sexual health and risky behaviours

Variable	*n*	%
Had sex at a festival following drug use		
Yes	477	39.98
No	716	60.02
Had unprotected sex		
No	262	21.96
Yes	316	26.49
Had unprotected sex and sought STI test		
No	1148	96.23
Yes	45	3.77
Had unprotected sex and sought information on sexual health		
No	1185	99.33
Yes	8	0.67
Had unprotected sex and sought the morning after pill		
Yes	23	1.93
No	1170	98.07

Due to rounding errors, percentages may not equal 100%

Most participants (84%/*n* = 1003) engaged in some form of harm reduction when taking drugs at festivals. More than three-quarters (76%/*n* = 901) stayed hydrated, almost two-thirds (62%/*n* = 704) took a test dose; however, only a small number (15%/*n* = 1 84) used one drug at a time. Table 3 illustrates harm-reduction practices that respondents suggested they engaged in.

**Table 3:** Harm-reduction practices in the last 12 months

Variable	*n*	%
Engaged in harm reduction	1003	84.07
Mono drug use	184	15.42
Stayed hydrated	901	75.52
Took test dose	704	59.01
Left time between doses	740	62.03

Due to rounding errors, percentages may not equal 100%

Of the 13% (*n* = 136) who stated they engaged in ‘other’ harm-reduction strategies not listed as an option on the survey, almost half (48%/*n* = 35) referred to the importance of their peer group, using together and the expertise within their group. Responses varied from ‘not using alone’, buddy systems, allocating a ‘sober friend’, telling friends what you have taken and asking friends for their experience with a particular batch and with drug use in general ‘I only do it with friends who are experienced’. As well as utilising peer groups for support, a small number of respondents reported the use of self-reporting test kits (*n* = 23), researching drugs online via sites such as ‘pill reports’ (*n* = 18) and using vitamins and supplements to support ‘serotonin recovery’ (*n* = 8).

More than one-fifth (22%/*n* = 263) of participants reported being unwell at a festival in the past, yet only one in five of this cohort sought help (21%/*n* = 54). Also, 16% (*n* = 192) said that they would not seek medical help if they needed it in the future. Nevertheless, the use of the term ‘unwell’ may have to be construed as vague; thus, with hindsight asking participants to rate, the severity of how they felt would be a much better metric. Participants’ comments were quite varied on the subject: ‘only if they were seriously unwell’ was frequently cited with fear of legal retribution being the primary reason for not seeking help.

Overwhelmingly, participants reported a willingness to engage with drug-checking services (Table 4). The vast majority (96%/*n* = 1149) would utilise onsite services, while 70% (*n* = 839) would engage with community-based services and more than three-quarters (75%/*n* = 897) reported that they would use an ‘amnesty bin’ for drugs if it were part of an alert system to notify if dangerous drugs are in circulation. Table 4 illustrates participants’ attitudes towards harm-reduction services should they be available at festivals.

**Table 4:** Attitudes towards harm-reduction services should they be available

Variable	*n*	%
Would utilise drug information if available	1015	85.08
Would avail of free condoms at a festival	946	79.30
Would utilise drug testing services at a festival	1149	96.31
Would utilise drug testing service (offsite)	839	70.33
Would you utilise an amnesty bin at a festival if part of an alert system	897	75.19

Due to rounding errors, percentages may not equal 100%

We were interested in whether or not age was a significant factor in whether respondents would utilise drug testing services either at festivals or offsite. We divided the respondents into two groups, 18–24 years old and 25 + years old.

A Pearson’s chi-square test (Table 5) was conducted to examine whether age and utilisation of drug safety testing service a festival were independent. The results were not significant based on an alpha value of *p* > 0.05, suggesting that age and utilisation of drug safety testing services at a festival could be independent of one another. However, there is a significant *p* < 002 gender (the other gender group was less than 5 in all cells, thus were removed from the analysis) difference for offsite testing; results suggest that males seem more likely to use this offsite service. Moreover, when all cases are taken together, the difference between testing modalities (onsite, offsite and amnesty bin) shows a significant difference *p* < 001 between those who would use onsite and offsite drug testing facilities.

**Table 5:** Attitudes towards drug checking services

			**On-site drug testing**				Total			
			No	Yes						
Gender_Cat	Female		21	520			541			
	Male		22	625			647			
Total			43	1145			1188			
**Chi-square tests**										
	**Value**		***Df***	**Asymptotic significance (2-sided)**			**Exact Sig. (2-sided)**		**Exact Sig. (1-sided)**	
Pearson chi-square	.196^a^		1	0.658						
Continuity correction^b^	0.082		1	0.775						
Likelihood ratio	0.195		1	0.659						
Fisher’s exact test							0.756		0.386	
Linear-by-linear association	0.196		1	0.658						
No. of valid cases	1188									
**Crosstab**										
**Count**										
			**Drug testing centre** **(offsite)**				**Total**			
			**No**	**Yes**						
Gender_Cat	Female		183	358			541			
	Male		168	479			647			
Total			351	837			1188			
**Chi-square tests**										
	**Value**		***Df***	**Asymptotic significance (2-sided)**			**Exact Sig. (2-sided)**		**Exact Sig. (1-sided)**	
Pearson chi-square	8.745^a^		1	0.003						
Continuity correction^b^	8.371		1	0.004						
Likelihood ratio	8.724		1	0.003						
Fisher’s exact test							0.003		***0.002	
Linear-by-linear association	8.738		1	0.003						
No. of valid cases	1188									
**Crosstab**										
**Count**										
			**Amnesty bin_drug drop**				**Total**			
			**No**	**Yes**						
Gender_Cat	Female		131	410			541			
	Male		164	483			647			
Total			295	893			1188			
**Chi-square tests**										
	**Value**		***Df***	**Asymptotic significance (2-sided)**			**Exact Sig. (2-sided)**		**Exact Sig. (1-sided)**	
Pearson chi-square	.203^a^		1	0.653						
Continuity correction^b^	0.147		1	0.702						
Likelihood ratio	0.203		1	0.652						
Fisher’s exact test							0.686		0.351	
Linear-by-linear association	0.203		1	0.653						
No. of valid cases	1188									
**On-site drug testing × drug testing drug centre cross-tabulation**										
					**Drug testing drug centre**			**Total**		
					**No**	**Yes**				
On-site drug testing		No			34	10		44		
		Yes			320	830		1150		
Total					354	840		1194		
**Chi-square tests**										
		**Value**			***df***	**Asymptotic significance (2-sided)**		**Exact Sig. (2-sided)**		**Exact Sig. (1-sided)**
Pearson chi-square		49.676^a^			1	0.000				
Continuity correction^b^		47.334			1	0.000				
Likelihood ratio		44.394			1	0.000				
Fisher’s exact test								0.000		
Linear-by-linear association		49.634			1	0.000				
No. of valid cases		1194								

^a^Zero cells (0.0%) have an expected count of less than 5. The minimum expected count is 159.84

^b^Computed only for a 2 × 2 table

The average age of participants was 24 years old, with the majority identifying as either employed or studying full time. The most frequently observed category for procurement of drugs was a ‘known source or dealer (73%/*n* = 874) and friend (55%/*n* = 657). Overwhelming participants said they did not procure drugs online (95%/*n* = 1131).

## Discussion

The current study is the first of its kind in an Irish context. The study provides critical insights into drug-use patterns and harm-reduction practices as well as attitudes towards drug-checking services among individuals who attend music festivals in Ireland. Festival drug use is a serious concern [14, 17]. For the cohort who responded to the survey, drug use was a significant part of the festival experience, with 96% of the current cohort reporting use. Moreover, of those who reported drug use, 86.9% suggested that they engaged in polysubstance use on average participants reported using three substances. The most commonly cited combinations were alcohol and cocaine, most likely combined with ketamine and MDMA.

Drugs which are not represented in general population data in Ireland such as ketamine (63%) and 2 CB (26%) were more commonly reported in this study than more well-known substances such as amphetamine (17%). Although low levels of psychedelics (mushrooms, 14% and DMT 2%) and NPS (2%) are recorded in Ireland, the upward trend towards higher use of mushrooms, 2 CB, DMT, nitrous oxide and mephedrone while attending festivals abroad is interesting. These responses could be an indicator of use among other sub-populations or of use occurring in other settings, outside of the festival arena. These findings provide a rationale for further investigation into drug-using cohorts not presenting to traditional addiction services.

The findings of the study suggest that similar to their European and Australian counterparts, Irish festival attendees tend to procure drugs through a ‘known source’ or a friend, thus further blurring the lines between a social exchange, distribution and dealing [18, 19]. Unlike in previous studies, the festival attendees surveyed relied less on the internet as a means of procurement and exchange. This study supports EU reports indicating low levels of online sourcing in Ireland through the darknet; however, further research is required in relation to this area and the use of the surface web and mobile applications. This pattern may have shifted as a result of the COVID-19 pandemic.

The current cohort reported high levels of engagement with and knowledge of a variety of harm-reduction practices. Practices such as starting slowly with smaller amounts and incremental consumption have shown to be effective strategies to minimise health-related harms with similar populations [20, 21]. There is limited literature on the proportion of recreational drug users who engage in harm-reduction strategies and what they define as a harm-reduction strategy. Therefore, the current findings serve to help address this issue. Fernandez-Calderon et al. (2014) found that individuals who identified as frequent polysubstance users engaged in less harm-reduction practices. Contrary to these findings, participants in the current study, despite engaging in high rates of polysubstance use, still frequently engaged in harm-reduction practices.

One worrying aspect of the current study was that, despite needing medical support, only one in five festival attendees sought it. The threat of legal retribution appears to be deterring individuals from seeking help. Thus, the presence of law enforcement at festivals may unintentionally increase the health risks associated with drugs used in this setting. In addition to the health risks associated with their drug use, 26.5% of the current cohort also engaged in riskier sexual behaviours. Further corroborating the findings of Lim et al. (2007), the authors found that 30% of the participants who engaged in sexual activity within the last year did not use condoms. The utilisation of support services following unprotected sex at festivals for the current cohort was also worryingly low.

Drug checking is a public health intervention that may provide an opportunity for harm reduction and positively influence festival attendees. The findings of this study suggest that this cohort is eager to engage with drug testing, mainly onsite, community-based and warning mechanisms linked with ‘amnesty bins’, should they be provided in Ireland. Given the opportunity, such services can access new user populations that would otherwise not be in reach [14]. Moreover, such services have been integral in reducing harm by detecting adulterants and alerting festival attendees and medical and support personnel, as well as informing existing national early warning systems [22]. Murphy et al. 2021 highlight how friends are most likely to influence the utilisation a drug checking service, illustrating the significance of peers in influencing norms, practices and behaviours [13]. The role of peers as educators and influencers needs further exploration. There is growing evidence to support the use of peers to influence practices and behaviours around drug use [23].

In her study, Measham (2019) piloted the first onsite drug-checking service in the UK. The pilot program was successful in diminishing risk onsite and using face-to-face time with festival attendees to offer harm-reduction advice. Based on these consultations, one-sixth adjusted their consumption. More recently, Measham (2020) examined the feasibility of community-based drug-checking services in the UK. The current finding that males are more likely to engage with community service may help when attempting to target this group. Community-based services arguably have better conditions for intervention not just for drug use but also for safe sexual health practices, service users are less likely to be intoxicated, and services can reach other vulnerable drug users who are not likely to attend festivals.

Participants were asked about drug use in the last 12 months. However, the frequency of drug use was omitted, which may have provided valuable information about a specific subgroup of participants. Grouping less frequent or one-time drug users may account for the significant difference between drug users’ and non-drug users’ attitudes towards the provision of drug-checking services at festivals. Nevertheless, a key objective of this research was to determine whether drug users would utilise drug-checking services at festivals. As such, all drug users must be considered, irrespective of the frequency of their use. Similarly, we asked participants about their utilisation of drug-checking facilities as harm-reduction measures but did not ask whether the results would influence their drug use.

Although drug checking is not the only solution to reducing harm and mortality at festivals, it does present a strong, evidence-based harm-reduction strategy [13–15] that warrants a trial in Ireland and the evidence from this survey indicates that attendees in Ireland would be open to utilising this approach. Public health messages have prominently featured warnings regarding the changing contents and potency of drugs since the emergence of NPS onto the Irish market a number of years ago. More recently, there has been an increase in NPS benzodiazepines appearing in illicitly sourced tablets. Moving forward, the provision of drug identification services could support surveys to ensure the accurate reporting and monitoring of emerging drug trends in nightlife settings which could help inform early harm-reduction responses.

Emergency critical medical care requires further expert discussion to consider the management and preparedness for critical illness, toxidromes and hyperpyrexia at events. Policies and protocols need to be developed to consider medical responses, staff provision and the development of a risk matrix to inform decisions concerning resources deployed at events. Perhaps the most critical action in the immediate is to develop a robust public health policy and campaign ensuring those who use drugs will seek help when needed without consequence or retribution. This requires a firm policy developed with key stakeholders such as festival-goers, event organisers, medical experts and key decision-makers from criminal justice.

## Strengths and limitations

A significant strength of this study is that it is, to our knowledge, the first extensive national survey of festival attendees to measure drug trends and related behaviours and attitudes towards drug-checking services. Most previous surveys have focused on a single event.

A limitation of this study is that it relied on self-reported data. To reduce the likelihood of safety hazards and inaccurate reporting associated with the intoxication of survey participants, surveys were conducted during festival season but not at festivals. Convenience sampling could also lead to systematic bias when comparing the findings to other populations of drug users and the general population. Importantly, attitudes towards using drug-checking services and amnesty bins do not necessarily translate to behaviour.

## Conclusion

Reducing harm from drug use is a critical public health issue, and our research provides valuable insight into the attitudes and behaviours of a high-risk and under-consulted population. The fact that the evidence from this survey indicates that those young people who use drugs at festivals would be prepared to utilise drug checking services and amnesty bins should help inform the Public Health response to this important area.
